# Hippocampal NMDA receptors and anxiety: At the interface between cognition and emotion

**DOI:** 10.1016/j.ejphar.2009.10.014

**Published:** 2010-01-10

**Authors:** Christopher Barkus, Stephen B. McHugh, Rolf Sprengel, Peter H. Seeburg, J. Nicholas P. Rawlins, David M. Bannerman

**Affiliations:** a Department of Experimental Psychology, University of Oxford, South Parks Road, Oxford, OX1 3UD, UK; b Max-Planck Institute of Medical Research, Department of Molecular Neurobiology, D-69120 Heidelberg, Jahnstrasse 29, Germany

**Keywords:** Spatial learning, Synaptic plasticity, Lesion, Dorsal hippocampus, Ventral hippocampus, Dentate gyrus

## Abstract

David De Wied had a fundamental interest in the brain and behaviour, with a particular interest in the interface between cognition and emotion, and how impairments at this interface could underlie human psychopathology. The NMDA subtype of glutamate receptor is an important mediator of synaptic plasticity and plays a central role in the neurobiological mechanisms of emotionality, as well as learning and memory. NMDA receptor antagonists affect various aspects of emotionality including fear, anxiety and depression, as well as impairing certain forms of learning and memory. The hippocampus is a key brain structure, implicated in both cognition and emotion. Lesion studies in animals have suggested that dorsal and ventral sub-regions of the hippocampus are differentially involved in dissociable aspects of hippocampus-dependent behaviour. Cytotoxic lesions of the dorsal hippocampus (septal pole) in rodents impair spatial learning but have no effect on anxiety, whereas ventral hippocampal lesions reduce anxiety but are without effect on spatial memory. This role for the ventral hippocampus in anxiety is distinct from the role of the amygdala in other aspects of emotional processing, such as fear conditioning. Recent studies with genetically modified mice have shown that NR1 NMDA receptor subunit deletion, specifically from the granule cells of the dentate gyrus, not only impairs short-term spatial memory but also reduces anxiety. This suggests that NMDA receptors in ventral hippocampus may be a key locus supporting the anxiolytic effects of NMDA receptor antagonists. These data support Gray's neuropsychological account of hippocampal function.

## 1 Introduction

The N-methyl-d-aspartate (NMDA) subtype of glutamate receptor is an important mediator of synaptic plasticity, reflecting (i) its dual ligand and voltage gating, (ii) its calcium conductance and (iii) the temporal kinetics of its channel activity (Kew and Kemp, 2005). NMDA receptor antagonists prevent the induction of certain forms of long-term potentiation, long-term depression and depotentiation (Collingridge et al., 1983; Dudek and Bear, 1992; Fujii et al., 1991; Mulkey and Malenka, 1992). The NMDA receptor also plays a central role in both emotionality and cognition. NMDA receptor antagonists affect various aspects of emotionality including fear, anxiety and depression, as well as impairing certain forms of learning and memory. Brain structures in the medial temporal lobe, such as the hippocampus and amygdala, lie at the interface between cognition and emotion, and are likely candidate sites at which NMDA receptor antagonists might exert their effects on emotionality. In particular, NMDA receptors in the hippocampus may be the key locus for the anxiolytic effects of these drugs. In this article we will first review the effects of NMDA receptor antagonists on anxiety. We will then review the evidence, both from lesion studies in animals and from human brain imaging, suggesting that the hippocampus (and in particular the ventral hippocampus) and amygdala make dissociable contributions to anxiety and fear respectively. Finally, we will discuss recent evidence from genetically modified mice which specifically implicates NMDA receptors in the hippocampus in anxiety.

## 2 NMDA receptor antagonists and emotionality

There is now an extensive literature describing the effects of NMDA receptor antagonists on emotional processing in rodents. Broadly speaking, these studies have often been divided along the lines of tests of either (i) unconditioned anxiety, or (ii) conditioned fear, although, as we shall discuss later in this article, this distinction may be over-simplistic. Nevertheless, for the purposes of briefly reviewing the literature on NMDA receptor antagonists we will keep to this distinction, and we will focus primarily on tests of unconditioned anxiety.

### 2.1 NMDA receptor antagonists and unconditioned anxiety

A number of different classes of NMDA receptor antagonists, acting at different sites on the NMDA receptor complex, have been assessed using unconditioned tests of anxiety in rodents. For example, several competitive NMDA receptor antagonists have been shown to exhibit anxiolytic effects in various different laboratory tests of anxiety. For example, 3-(2-carboxy piperazine-4yl)-propyl-1-phosphonic-acid, (CPP) increased the amount of time spent in the open arms of the elevated plus maze and increased social interaction in rats, consistent with a reduction in anxiety (Corbett and Dunn, 1993; Dunn et al., 1989). Dunn et al. (1989) also investigated the effects of two related competitive antagonists, AP5 (2-amino-5-phosphonoheptanoate) and AP7 (2-amino-7-phosphonoheptanoate), with both compounds producing anxiolytic effects on the elevated plus maze and increased social interaction. Subsequent studies showed that intracerebroventricular (i.c.v.) injections of AP7 in rats were also effective in reducing anxiety on the elevated plus maze (Plaznik et al., 1994).

Possibly the best characterised of all of the NMDA receptor antagonists in the laboratory is the non-competitive channel blocker, MK-801 (dizocilpine), which is an effective anxiolytic in conflict intake studies (Corbett and Dunn, 1993; Jessa et al., 1996; Plaznik et al., 1994; Soderpalm et al., 1995; Xie and Commissaris, 1992), on the elevated plus maze (Bertoglio and Carobrez, 2003; Corbett and Dunn, 1991, 1993; Criswell et al., 1994; Dunn et al., 1989; Engin et al., 2009; Fraser et al., 1996; Karcz-Kubicha et al., 1997; Plaznik et al., 1994; Wieronska et al., 2003), and in tests of social interaction in the rat (Corbett and Dunn, 1991, 1993; Dunn et al., 1989). Other non-competitive NMDA receptor antagonists have also exhibited anxiolytic effects in rodents. For example, phencyclidine (PCP) reduces anxiety in rat conflict intake studies (Porter et al., 1989) and on the elevated plus maze in mice (Wiley et al., 1995). Surprisingly, ketamine, another widely studied non-competitive NMDA receptor antagonist, produced anxiogenic effects in rats. Ketamine decreased time spent in the open arms of the elevated plus maze and decreased social interaction (Silvestre et al., 1997, but see also Engin et al., 2009). This effect on the elevated plus maze was also seen following chronic (35 day) oral voluntary consumption (Silvestre et al., 2002), but not when testing weeks after five daily injections of 30 mg/kg i.p (Becker et al., 2003). In contrast, ketamine has been shown to be an anxiolytic on the elevated plus maze in mice (Hayase et al., 2006). Therefore, the animal data on ketamine is contradictory and is complicated by the possibility that the drug might exert some of its effects through other receptor classes (see for instance Abelson et al., 2006; Chen et al., 2009; Hevers et al., 2008; Hirota and Lambert, 1996; Hirota et al., 1999; Kapur and Seeman, 2002; Maeng et al., 2008; Okamoto et al., 2003). Interestingly, at doses as low as 0.1 mg/kg i.v., ketamine reduces anxiety in healthy human volunteers (Krystal et al., 1994), consistent with the anxiolytic profile displayed by other competitive and non-competitive NMDA receptor antagonists in rodents.

Antagonists acting at the glycine site on the NMDA receptor complex also produce anxiolytic effects in rodents, These include 7-chlorokynurenic acid (7 CKA, Anthony and Nevins, 1993; Trullas et al., 1989), 5,7-dichlorokynurenic acid (5,7 DCKA, Corbett and Dunn, 1993; Kehne et al., 1991; Plaznik et al., 1994) and L-701,324 (Karcz-Kubicha et al., 1997; Kotlinska and Liljequist, 1998; Przegalinski et al., 1998). In addition, ifenprodil, an antagonist acting at the polyamine site on the NR2B subunit, has also been shown to reduce anxiety on the elevated plus maze in mice (Fraser et al., 1996). These results demonstrate that other modulatory sites on the NMDA receptor complex may be targeted to alter anxiety in addition to direct antagonism of the NMDA pore region or the glutamate binding site.

Thus, there is considerable evidence that NMDA receptor antagonists, acting at a number of different sites on the receptor complex, can reduce anxiety, both in humans and experimental animals. But where do these drugs act to produce their anxiolytic effects, and what are the neurobiological mechanisms involved? The majority of drug studies have involved systemic administration and so it is difficult to establish the locus of effect, and to attribute changes in behaviour to a particular mechanism. Recent advances in transgenic mouse technology have allowed specific receptor subunits to be targeted in specific brain regions. We recently studied the behaviour of genetically modified mice in which the NR1 subunit of the NMDA receptor was deleted specifically from the granule cells of the dentate gyrus and found evidence for a role for hippocampal NMDA receptors in both learning and anxiety (Niewoehner et al., 2007). Before describing the behavioural phenotype in these animals it is first worth discussing why the hippocampus is considered an important brain area in emotionality, and, in particular, for anxiety.

## 3 The hippocampus and behaviour

The predominant view in neuroscience is that the hippocampus is involved in memory. This stems originally from studies of human amnesics with hippocampal damage, such as H.M. (Scoville and Milner, 1957), and since this memory loss in human patients was first described, numerous mnemonic theories of hippocampal function have been put forward, and have dominated the field of hippocampal research. Rodents with complete hippocampal lesions are dramatically impaired on tests of spatial learning such as the Morris watermaze task (Deacon et al., 2002a; Morris et al., 1982, 1990), but hippocampal lesions also produce clear effects on certain non-spatial memory tasks, particularly when there is a temporal component (Fortin et al., 2002; Kesner et al., 2002; Mariano et al., 2009; Meck et al., 1984).

However, in recent years there has been an increasing interest in the role of the hippocampus in emotionality. If one types “hippocampus” into PubMed along with various other keys words (as of May, 2009), then, not surprisingly, there are an enormous number of papers concerning “memory” (13,755). But there is also huge interest in this brain structure in the context of emotionality. For example, “hippocampus” and “emotion” gets over 2000 hits, “hippocampus” and “anxiety” gets 1600 hits, “hippocampus” and “depression” gets nearly 5000 hits, and “hippocampus” and “stress” identifies over 5000 papers. So there is a significant interest in the role of the hippocampus in emotion and this has came to the fore in recent years with the suggestion that anti-depressant drugs might produce their clinical effects on emotion by increasing the production of new neurons in the dentate gyrus subfield of the hippocampus (Santarelli et al., 2003). Although the precise relationship between hippocampal neurogenesis and emotionality remains unclear, and more recent studies have failed to substantiate fully the requirement for hippocampal neurogenesis in anti-depressant drug action (David et al., 2009; Holick et al., 2008; Huang et al., 2008), these studies nevertheless highlight a growing interest in the hippocampus in aspects of emotion, and, in particular, in anxiety and depression. Importantly, hippocampal lesions also reduce anxiety on a variety of laboratory tests (Deacon et al., 2002b; Gray, 1982; Gray and McNaughton, 1983; Gray and McNaughton, 2000).

Thus, the hippocampus is important not only in certain kinds of memory, but also for aspects of emotional processing, and this potential diversity of function is illustrated by the range of behavioural effects that are observed after hippocampal lesions in rodents. In recent years it has become clear that these different effects of hippocampal lesions involve different sub-regions of the hippocampus.

### 3.1 Dorsal hippocampal lesions impair spatial learning

The introduction and widespread deployment of cytotoxic fibre-sparing lesions was a key development because it allowed the contributions of the different sub-regions within the hippocampus to different aspects of hippocampus-dependent behaviour to be identified. Cytotoxic lesions, made by injecting glutamate analogues such as NMDA or ibotenic acid, destroy the cells in the region of interest but spare overlying tissue, fibres of passage and blood vessels in that area (Jarrard, 1989). They therefore allow the experimenter to selectively lesion one hippocampal sub-region without affecting the other. In a series of experiments we compared the behavioural effects of hippocampal lesions, comprising either the dorsal 50% (i.e. from the septal pole, corresponding to the posterior hippocampus in primates) or the ventral 50% (i.e. from the temporal pole, corresponding to the anterior hippocampus in primates).

In agreement with the previous work of Moser et al. (1993, 1995), we found that the dorsal hippocampus was essential for spatial learning on tasks including the Morris watermaze (Bannerman et al., 2002, 1999; McHugh et al., 2004). In contrast, ventral hippocampal lesions were without effect on spatial memory (see also Bannerman et al. (2003). For example, the standard version of the watermaze task involves training rodents to find a hidden escape platform that remains in a fixed location on every trial (Morris et al., 1982). The rat is released from various different starting points around the perimeter of the maze and has to use the extramaze spatial cues located around the room to find the platform. Rodents with complete or dorsal hippocampal lesions had longer latencies and travelled greater distances to find the platform during training compared to sham operated control animals (Bannerman et al., 1999). Furthermore, when a probe test was conducted, during which the platform was removed from the pool, controls spent most of their time searching in the area of the pool where the platform was located, whereas rats with complete or dorsal hippocampal lesions showed no such preference for the training quadrant. In contrast, the ventral hippocampal lesioned rats were indistinguishable from controls, both during training and the probe test.

This dissociation between the effects of dorsal and ventral hippocampal lesions has also been observed on other spatial memory tasks, including the spatial working memory, T-maze rewarded alternation task (Bannerman et al., 2003, 1999; Hock and Bunsey, 1998) and the radial maze (Pothuizen et al., 2004). Interestingly, there are parallels in the human literature. Famously, it has been shown that London taxi drivers, a subset of the population that are continuously engaged in complex spatial navigation as part of their daily work, possess enlarged hippocampi compared to age-matched controls, and this increase is specific to the posterior hippocampus (the primate equivalent of the rodent dorsal hippocampus, Maguire et al., 2000).

### 3.2 Ventral hippocampal lesions reduce conditioned freezing

So what does the ventral hippocampus do? The nature of the anatomical connections to and from the ventral hippocampus may provide a clue to its function. The ventral sub-region differs markedly from the dorsal sub-region in its anatomical connections (for reviews see Dolorfo and Amaral, 1998; Krettek and Price, 1977; Moser and Moser, 1998). It projects to the prefrontal cortex and is closely connected to the bed nucleus of the stria terminalis (BNST) and the amygdala, as well as other sub-cortical structures which are associated with the hypothalamic-pituitary-adrenal (HPA) axis. This strong connectivity between ventral hippocampus and both the hypothalamus and the amygdala, made it tempting to propose a role for the ventral sub-region in aspects of emotionality.

Emotional processing is widely studied using simple, Pavlovian fear conditioning procedures. A neutral, discrete stimulus such as a tone (the conditioned stimulus; CS) is paired with a motivationally significant stimulus, usually a footshock (the unconditioned stimulus; US). After several CS–US pairings, presentation of the tone CS evokes an increase in freezing (the conditioned response; CR), in the absence of any footshock, suggesting that the animal has learned an association between the two stimuli (Bouton and Bolles, 1979). In addition, the animal also learns to associate the experimental context with the footshock and is therefore likely to freeze on being returned to the operant chamber in which the conditioning took place, in the absence of any other stimuli.

The neurobiology of fear conditioning has been studied extensively and the importance of the amygdala for fear conditioning is well established, both from lesion studies in animals (for example Phillips and LeDoux, 1992), and from human imaging studies (for example Buchel et al., 1998; Hasler et al., 2007; LaBar et al., 1998). In addition, hippocampal lesions can also disrupt conditioned freezing, although it has often been reported that the deficits in freezing are specific to the experimental context and do not occur with punctate CS cues, such as a light or tone (Kim and Fanselow, 1992; Phillips and LeDoux, 1992) (but see also (Good and Honey, 1997).

Consistent with a preferential role for ventral hippocampus in emotionality, we found deficits in conditioned freezing in ventral, but not dorsal, hippocampal lesioned rats (Bannerman et al., 2003; Maren, 1999; Maren et al., 1997; Richmond et al., 1999). In our study, ventral hippocampal lesioned rats, and rats with complete hippocampal lesions, exhibited reduced freezing responses relative to sham-operated controls immediately after the delivery of footshocks during the conditioning session, and also during a subsequent extinction session when animals were returned to the experimental context in the absence of further shocks (Richmond et al., 1999). In addition, we also saw reduced freezing to the tone CS, presented in a separate, novel context, in both ventral and complete lesion groups, suggesting that the deficits in conditioned freezing are not always specific to freezing induced by the experimental context. Importantly, these results provided a double dissociation between the effects of ventral hippocampal damage on conditioned freezing and the effects of dorsal hippocampal lesions on spatial learning, thus supporting a specific role for the ventral sub-region of the hippocampus in emotionality (Bannerman et al., 2004).

### 3.3 The role of the hippocampal formation in anxiety

Neuropsychological accounts of the role of the hippocampus in emotionality have emphasized its importance for anxiety (Gray, 1982; Gray and McNaughton, 2000). Furthermore, these accounts suggest that anxiety is the emotional response that arises in situations of conflict and uncertainty. An obvious source of conflict or uncertainty arises when there is a mismatch between what is expected on the basis of information retrieved from memory and what actually happens. Human neuroimaging studies have implicated the hippocampal formation in the detection of mismatches between expected and actual experience during fear conditioning. Ploghaus et al. (2000) used fMRI to study brain activation using a fear conditioning paradigm in which different coloured lights were associated with either no stimulation, delivery of a non-noxious warm stimulus or a painful heat stimulus applied to the back of the hand. Activation of the hippocampal formation (hippocampus and parahippocampal gyrus/entorhinal cortex) was associated with three different situations during the fear conditioning paradigm, (i) novel, unexpected pain when subjects had no prior knowledge and hence no expectation as to which visual stimulus was paired with pain, (ii) unexpected omission of pain when the expected painful stimulus was absent during extinction, and (iii) counter-expected pain when the painful stimulus was now delivered when no stimulus was expected (i.e. the painful stimulus was delivered unexpectedly during a signalled rest or safety period indicated by a different background contextual cue). Therefore, hippocampal activation was associated with both unexpected delivery and unexpected omission of painful stimuli.

More recently, hippocampal activation has also been observed during mismatch detection in human fMRI studies, using non-aversive paradigms (Kumaran and Maguire, 2006, 2007). In parallel, hippocampal lesioned rats failed to detect mismatches that were generated when an auditory stimulus that had been specifically associated with one visual stimulus was presented with a different, but equally familiar, visual stimulus using a non-aversive testing paradigm (Honey et al., 1998). Rats were trained with two audio–visual sequences. In the first sequence a tone was always followed by a constant light whereas for the second sequence a clicker was always followed by a flashing light. Both sham and hippocampal lesioned animals habituated to the visual stimuli as training continued. On a test trial, the auditory stimuli preceding the visual target stimuli were switched (i.e. clicker-constant light and tone-flashing light). This resulted in renewed orienting responses to the visual stimuli in the control subjects, suggesting that they had detected the associative mismatch, but this was not the case in the hippocampal lesioned animals.

In a recent human PET imaging study, brain activation was studied in anticipation of electric shocks that were either predicted by a visual cue or delivered unpredictably and thus associated with the background context (Hasler et al., 2007). Anterior (i.e. ventral) hippocampal activation was associated with the unpredictable, contextual threat condition. Of course, this hippocampal activity could reflect a role in processing contextual information (Hirsh, 1974), but it could also reflect the increased uncertainty associated with the context as a predictor of shock. The importance of uncertainty in generating anxiety and hippocampal activation has been demonstrated in a study by Ploghaus et al. (2001) in which they studied the exacerbation of pain by anxiety. A visual stimulus (V1) was associated with a moderate intensity, painful stimulus on all trials and thus generated low anxiety. A second visual stimulus (V2) was paired with a moderate pain stimulus on the majority of trials, but on a small number of trials preceded the delivery of a high intensity painful stimulus. This resulted in a high anxiety condition, with subjects reporting feelings of high anxiety on presentation of V2, prior to the delivery of the painful US. V2 also produced a larger response to the moderately painful stimulus in terms of pain ratings (anxiety-induced hyperalgesia), and increased neuronal activity in an area of the parahippocampal gyrus, corresponding to the entorhinal cortex. Thus, the anxiety induced by uncertainty can also result as a consequence of an unpredictable association between a punctate visual CS and the magnitude of an aversive US.

The importance of uncertainty in generating hippocampus-dependent anxiety may explain why hippocampal lesion studies in rodents have often found what appear to be specific deficits in contextual fear conditioning, with no effect on conditioned freezing to punctate auditory or visual cues. By its very nature, the context is going to be a relatively poor predictor of the delivery of the footshock (i.e. for the vast majority of the time that the animal is experiencing the experimental context it is not receiving shock). In contrast, there is usually an absolute relationship between the tone or the light CS and the delivery of the footshock, making the punctate cue a perfect predictor of shock. Therefore, the context will generate much greater uncertainty in terms of its ability to predict the occurrence of the shock.

### 3.4 Hippocampal lesions and unconditioned tests of anxiety

There is considerable additional evidence from lesion studies in rodents that the hippocampus is important for anxiety. Hippocampal lesions and, specifically ventral hippocampal lesions, have robust effects on ethologically based, unconditioned laboratory tests of anxiety which generate an approach/avoidance conflict. Indeed, the approach/avoidance conflict provides the basis for the majority of laboratory anxiety tests. For example, the elevated plus maze, considered by many as the industry standard test of anxiety, generates a conflict between two concurrent goals that can potentially drive the behaviour of the animal. The animal has an exploratory drive to investigate the open arms of the elevated plus maze. Therefore, there is a potential approach response towards the open arms. Against this, the open arms are exposed and potentially dangerous (compared to the closed arms). Therefore, there is also a potential avoidance response towards the open arms. These concurrent approach and avoidance responses generate anxiety. Hippocampal lesioned animals display reduced anxiety and increased approach behaviour on simple, ethological, unconditioned tests like the elevated plus maze (Bannerman et al., 2002, 2003; Kjelstrup et al., 2002; McHugh et al., 2004, see also Chudasama et al., 2008). In this regard, the behavioural effects of hippocampal lesions closely resemble the effects of anxiolytic drugs such as benzodiazepines which also increase approach behaviour (Gray, 1982; Gray and McNaughton, 1983, 2000).

**Fig. 1:**
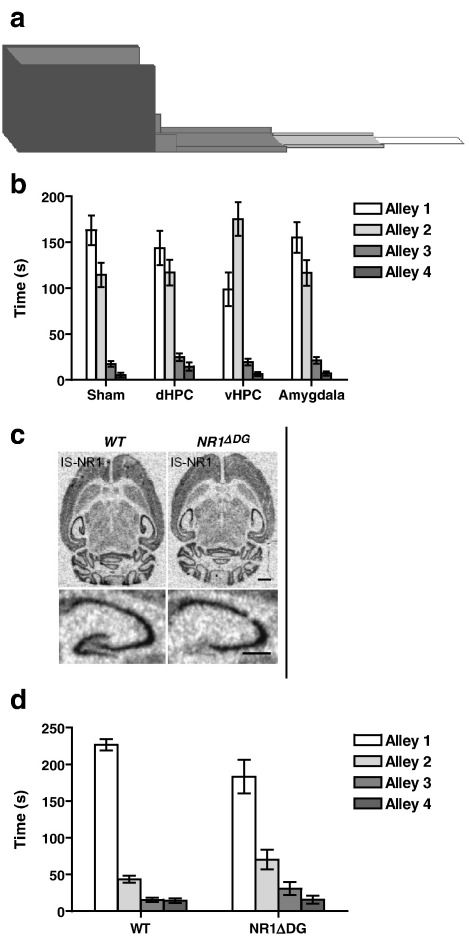
NMDA receptors in the ventral hippocampus mediate anxiety. (a) The successive alleys test of anxiety is a modified version of the elevated plus maze. The apparatus consists of four sections or alleys of increasing anxiogenic character in a linear arrangement. (b) The effects of sham, dorsal hippocampal (dHPC), ventral hippocampal (vHPC) and amygdala lesions on the successive alleys test in rats. vHPC lesioned rats were less anxious than all of the other three groups (e.g. time in Alley 2; *F* (3,47) = 4.0; *P* < 0.05, Duncan's pairwise comparisons at *P* < 0.05), (from McHugh et al., 2004). (c) *In situ* hybridisation of littermate control animals (left) and DG specific NR1 deleted mice (*NR1*^*ΔDG*^) (right) with NR1 specific probe. The lower panels give a zoom of the hippocampus from the respective upper panels (scale bars: 1 mm). (d) The effect of NR1 NMDA receptor subunit deletion from granule cells of the dentate gyrus on the successive alleys test in mice. *NR1*^*ΔDG*^ mice were less anxious than wild-type littermate controls, spending comparatively more time in the more anxiogenic alleys (genotype × alley interaction — *F* (2,66) = 3.4; *P* < 0.05, simple main effects; Alley 2 — *F* (1,88) = 4.1; *P* < 0.05, Alley 3 — *F* (1,88) = 4.0; *P* = 0.05) (from Niewoehner et al., 2007; Supplementary material).

For example, we assessed anxiety in groups of lesioned rats using the successive alleys test, which is a modified form of the elevated plus maze. The apparatus consists of four sections or alleys of increasing anxiogenic character in a linear arrangement (Fig. 1). The first section is fully enclosed with high walls and is painted black. By gradually reducing the height of the side walls and the width of the arms, and by increasing the brightness, Sections 2, 3 and 4 provide increasingly anxiogenic stimuli. The more anxious an animal, the more time it will spend in the enclosed arms, and the less time it will spend in the more open, exposed (and potentially dangerous) arms. The fact that there are 4 arms of differing anxiogenic character in a linear arrangement is designed to make the test more sensitive by providing a range of anxiogenic conditions. This arrangement also avoids the interpretational difficulties associated with the central square of the elevated plus maze. Animals were placed in the apparatus for 5 min and the times spent in each of the 4 sections were measured. Rats with dorsal or ventral hippocampal lesions, and amygdala lesioned animals were compared to sham-operated controls (McHugh et al., 2004). Whereas sham rats, rats with dorsal hippocampal lesions, and rats with amygdala lesions spent most of their time in the least anxiogenic arm, (Section 1), the animals with ventral hippocampal lesions spent comparatively more time in the more anxiogenic, Section 2 (see Fig. 1). This result was similar to the effects observed when normal rats were given a 2.5 mg/kg dose of chlordiazepoxide. Thus, animals with ventral hippocampal lesions showed reduced anxiety.

The same pattern of results was also observed on a variety of other anxiety tests, including the food neophobia (hyperneophagia) task and a social interaction task. Ventral hippocampal lesioned animals were faster to eat a novel foodstuff in a novel, potentially dangerous, environment (Bannerman et al., 2002; McHugh et al., 2004). They also displayed increased levels of social interaction compared to other groups when placed with an unfamiliar animal. In each case the ventral lesioned animals demonstrated behaviour consistent with a reduction in anxiety, resembling the effects seen with benzodiazepines.

Importantly, the effects of ventral hippocampal lesions were dissociable, not only from the effects of dorsal hippocampal lesions on spatial memory, but also from the effects of amygdala lesions. Notably, amygdala lesions did not affect performance on the successive alleys test (McHugh et al., 2004), and others have previously shown that amygdala lesions are also without effect on the elevated plus maze (Kjelstrup et al., 2002; Treit and Menard, 1997). They do, however, reliably impair fear conditioning (for example Phillips and LeDoux, 1992). This dissociation supports the idea that anxiety and fear are separate psychological constructs involving the hippocampus and amygdala respectively.

## 4 Fear versus anxiety

As previously suggested, anxiety arises when there is a conflict between potential response options available to the animal. In laboratory tests of anxiety, like the elevated plus maze or the successive alleys test, this manifests as an approach/avoidance conflict with respect to exploration of the open arms. It has been suggested that the distinction between fear and anxiety is best encapsulated by the concept of defensive direction (Gray and McNaughton, 2000). Whereas fear is a behavioural state that functions to remove the animal from a dangerous situation (active avoidance), or to deal with that dangerous situation by adopting the appropriate stimulus-specific response in the presence of a stimulus that has become associated with danger (such as freezing in response to a cue associated with footshock), anxiety functions to limit whether or not the animal should enter into a potentially dangerous situation (passive avoidance). In other words, fear is the response to a threat that is present, whereas anxiety is the response to a potential threat. These different behavioural responses to present or potential danger differentially depend on the amygdala and ventral hippocampus respectively, and exist within a hierarchical defense system that is arranged to protect the animal from harm.

Gray has suggested that the septo-hippocampal formation is the seat of anxiety in the brain, and that it acts, first, to detect situations of conflict or uncertainty, and then second, to resolve those conflicts and thus protect the animal from danger (and/or maximize its chances of reward; see Gray and McNaughton, 2000). It has long been suggested that the hippocampal formation could potentially act as a comparator, allowing novel or unexpected events to be detected by comparing the current state of the world with what would be expected on the basis of information retrieved from memory, and thus allow situations of conflict to be identified (Gray, 1982; Gray and McNaughton, 2000; Vinogradova, 1975). Having detected a conflict (a novel or unexpected event), the hippocampal formation acts to resolve the conflict by increasing levels of attention and arousal, and through behavioural inhibition of prior, on-going motor programs. These behavioural responses constitute anxiety and they allow the animal to gather more information in order to resolve the conflict before responding appropriately. Mechanistically, Gray and MacNaughton suggested that the hippocampal system resolves the conflict by increasing the weighting given to affectively negative information. In other words, in a normal animal the hippocampal system will act to favour avoidance behaviour over approach behaviour.

## 5 Hippocampal NMDA receptors and anxiety

It is therefore tempting to suggest that the anxiolytic effects of NMDA receptor antagonists reflect the blockade of NMDA receptors in the hippocampus, and, in particular, in the ventral hippocampus. Indeed, there is now evidence from a variety of sources to suggest that this is the case. For example, it is now possible to assess the importance of hippocampal NMDA receptors for anxiety using genetically modified mice in which particular receptor subunits can be specifically deleted from spatially restricted hippocampal subfields.

Using this approach we recently assessed the behaviour of mice that lack the NR1 subunit of the NMDA receptor exclusively from the granule cells in the dentate gyrus subfield of the hippocampus (Niewoehner et al., 2007, see also Fig. 1). In terms of electrophysiology, these mice exhibited normal LTP in the CA1 region but dramatically reduced LTP in both the medial and lateral perforant path inputs to the dentate gyrus. In terms of learning and memory, the dentate gyrus NR1 knockout mice exhibited a very selective impairment in short-term spatial working memory. Anxiety was assessed in these mice on a version of the successive alleys test that was modified for mice (Niewoehner et al., 2007, see Supplementary material). The dentate gyrus NR1 knockout mice were found to be less anxious. They were more likely to approach and explore the open, exposed sections of the apparatus compared to their wild-type littermates, thus resembling rodents with ventral hippocampal lesions (Fig. 1). More recently, we have also studied genetically modified mice that lack the NR2B subunit of the NMDA receptor, specifically from hippocampal granule and pyramidal cells in the dentate gyrus and CA1 subfields respectively (von Engelhardt et al., 2008). Again, these animals displayed a reduced anxiety phenotype on the successive alleys test, being more inclined to venture out into the more open sections of the maze compared to their wild-type littermates.

Therefore, studies with transgenic animals with hippocampal-specific NMDA receptor subunit deletions support a key role for hippocampal NMDA receptors in anxiety. However, at present these genetically modified mice are unable to differentiate between contributions from dorsal and ventral sub-regions of the hippocampus. To resolve along these lines a pharmacological approach is required. In a recent study, the NMDA receptor antagonist AP5 has been infused locally into either the dorsal or ventral hippocampus of rats prior to testing on the elevated plus maze (Nascimento Hackl and Carobrez, 2007). This study revealed an anxiolytic effect of 6 and 24 nmol AP5, but only when infused into the ventral hippocampus. There was no effect of the drug on measures of anxiety on the elevated plus maze when infused into dorsal hippocampus, using either AP5 (Nascimento Hackl and Carobrez, 2007) or the related compound AP7 (Padovan et al., 2000). This study thus provides evidence for a role for ventral hippocampal NMDA receptors in anxiety.

## 6 Conclusions

In summary, NMDA receptor antagonists exert a range of effects on emotionality, including a reduction of anxiety. In order to establish the neurobiological mechanisms and brain circuits that mediate these anxiolytic effects, it is necessary first to understand the psychological basis of anxiety, and to recognise that it represents a separate construct from other aspects of emotionality. Neuropsychological theories have suggested that anxiety is an emotional response that arises specifically in situations of conflict and uncertainty (Gray, 1982; Gray and McNaughton, 2000). Brain imaging studies in humans, and experimental lesion studies in animals, have implicated the hippocampus, and particularly, the ventral sub-region of the hippocampus, in anxiety. It is therefore tempting to suggest that NMDA receptor antagonists exert their anxiolytic effects through blockade of NMDA receptor in ventral hippocampus. Indeed, there is now evidence, both from studies with genetically modified mice in which NMDA receptor subunits are deleted specifically from the hippocampus, and from intra-hippocampal drug infusions in rats that this is the case.

The challenge now is to identify the NMDA receptor-dependent synaptic and cellular mechanisms that might underlie anxiety, and to establish if, and how, these same mechanisms might contribute to spatial memory performance. The internal anatomical structure within the hippocampus suggests a common algorithm being executed, both by the dorsal hippocampus in spatial memory and the ventral hippocampus in anxiety. For example, the same repeating lamellar structure exists along the entire dorso-ventral axis of the hippocampus, characterised by the well-defined tri-synaptic circuitry, through which information flows from entorhinal cortex to dentate gyrus to CA3 to CA1. This common architecture in both sub-regions suggests a common operation being performed in both spatial memory and anxiety. Studies with genetically modified mice in which NMDA receptors are selectively deleted from the different hippocampal subfields are likely to be of great importance for identifying this algorithm (or algorithms).
